# Referee Acknowledgement for 2011

**DOI:** 10.1038/bjc.2012.249

**Published:** 2012-06-05

In this issue, we publish the names of those who reviewed manuscripts for us in 2011.

The Editor-in-Chief, Specialist Editors and everyone involved in publishing *BJC* would like to extend our sincere thanks to them for contributing their expertise and time. Our referees play an invaluable role ensuring that *BJC* continues to publish the high-quality original papers and reviews that make it one of the world’s leading oncology journals.

Aaltonen, Lauri A

Aaronson, Neil

Abdollahi, A

Abe, Hiroyuki

Abe, Kenji

Abnet, Chrgistian

Adam, Liana

Adam, Rene

Adamo, Sergio

Adjei, Alex

Aertgeerts, Kathleen

Agalliu, Ilir

Agulnik, M

Ahlquist, David A

Ajani, Jaffer

Akiba, Suminori

Akiyama, Yoshimitsu

Aksoy, Sercan

Albanese, Chris

Al-Batran, Salah-Eddin

Albelda, Steven M

Albert, Réka

Aldape, Ken

Alexander, Dominik D

Alexander, G Caleb

Alexandroff, Anton

Alferez, Denis G

Allday, Martin

Allgayer, Heike

Al-Mulla, Fahd

Aloyz, Raquel

Alvares-Secord, Angeles

Amant, Frederic

Ambros, P

Amir, Ziv

Amoroso, Vito

Andrae, Bengt

Andre, Fabrice

Andreyev, H Jervoise N

Andrisani, Ourania

Antonelli, Alessandro

Antonsson, Annika

Aoki, Taku

Aparicio, Jorge

Arbuthnot, Patrick

Arbyn, Marc

Arias-Pulido, Hugo

Arkenau, Hendrik-Tobias

Arora, Shilpi

Arya, Arvind

Ashcroft, Margaret

Ashworth, Alan

Astrakianakis, George

Audisio, Riccardo

Auger, Jacques

Aune, Dagfinn

Aupérin, A

Autier, Philippe

Autorino, R

Ayadi, Wajdi

Bachmann, Jeannine

Badie, Christophe

Bae-Jump, Victoria L

Baguley, BC

Bai, Yidong

Baiocchi, Robert

Baker, JB

Bakkum-Gamez, Jamie N

Balkwill, Fran

Ballhausen, Wolfgang

Ballman, Karla

Balossier, Anne

Bamias, Aristotle

Banerjee, Debabrata

Bao, Ying

Barbera, Mariagnese

Barberi-Heyob, Muriel

Barbolina, Maria

Barker, Margo

Barresi, Valeria

Barron, Thomas I

Bartlett, David L

Bartolazzi, Armando

Barton, BE

Basile, JR

Batra, Raj K

Baudin, Eric

Bauerle, Tobias

Baxevanis, Constantin N

Beachy, PA

Begent, RHJ

Begg, Adrian

Behrens, Diana

Beier, CP

Beliën, JAM

Bellesso, Marcelo

Bell-McGuinn, Katherine M

Bellone, Graziella

Bellone, Stefania

Belshe, Robert

Bemis, Lynne

Ben Porath, Ittai

Benito, Manuel

Benjamin, R

Ben-Jonathan, Nira

Bennett, Charles

Benson, Al

Benson, Vicky

Beresford, Mark J

Berger, Adam C

Berger, Ann M

Berghmans, Thierry

Bernhardt, Peter

Bernstein, Mark

Berstein, Lev

Bertino, Joseph R

Bertolini, Francesco

Bevilacqua, Generoso

Bhargava, Rohit

Bhat, G Jayarama

Bhatt, Rupal

Bianchini, Giampaolo

Biggers, B

Bilici, Ahmet

Bindoli, Alberto

Bingle, Lynne

Birchmeier, Walter

Birmann, Brenda

Birner, Peter

Bíró, Tamás

Bischoff, Pierre

Bisdas, Sotirios

Blagden, Sarah P

Blaise Cook, Michael

Blanco, Ignacio

Blechacz, Boris

Bleiberg, Harry

Blettner, Maria

Bloemena, Elisabeth

Blonski, Wojciech

Blot, William

Bocca, Claudia

Boeck, Stefan

Boellaard, Ronald

Boerner, Julie

Bohlius, Julia

Boku, Narikazu

Boland, C Richard

Bold, Richard

Bonavida, Benjamin

Bonetti, A

Boni, Luigi

Bonnetain, F

Boothman, David

Borden, Ernest

Borg, Christophe

Borget, Isabelle

Borner, Markus

Bosari, Silvano

Bosch, Francesc

Bots, Michael

Boucheix, Claude

Boudreau, François

Bouvier, Anne-Marie

Bowen, Wayne

Bower, Mark

Boyd, Norman F

Boyle, Terry

Braakhuis, Boudewijn

Brabletz, Thomas

Bradbury, Penelope A

Bradley, Kevin

Braendengen, Morten

Brambilla, Elisabeth

Brasch, Robert C

Brasky, Theodore

Bray, Freddie

Breier, Albert

Bremner, Karen E

Brennan, Murray

Breton, Marie

Bria, Emilio

Bridgewater, John

Brody, Julia Green

Broggini, Massimo

Bromwich, Emma

Brouwers, Melissa

Brown, Jubilee

Brown, Nicola J

Brown, Robert

Brown, Stanley

Bruyand, Mathias

Buchler, Tomas

Buchsbaum, Donald

Buckstein, Rena

Budillon, Alfredo

Bueno-de-Mesquita, H Bas

Bukowski, Ronald

Bunnell, Craig

Burbos, Nikolaos

Burd, Randy

Burdon, Tom G

Burger, Maximilian

Burger, Robert

Burnett, Alan

Burnett-Hartman, Andrea N

Burris, Howard A

Butler, Lesley

Buyse, Marc

Byun, Seok Kyu

Cabibbo, Giuseppe

Cairney, Claire

Cairns, Benjamin

Calin, George

Calistri, Daniele

Calvani, Nicola

Campbell, Helen E

Campbell, Kerry

Campbell, Moray J

Campbell, Peter

Camps, C

Canevari, Silvana

Cantley, Lewis C

Canto, Maria

Canuto, Rosa

Cao, Guangwen

Capasso, M

Capitanio, Umberto

Cappocaccia, Riccardo

Cappuzzo, Federico

Carducci, Michael

Carneiro, Fatima

Carpino, Nicholas

Carson III, William

Carson, James A

Carvajal, RD

Cascinu, Stefano

Cassidy, Jim

Castanon, Alejandra

Castresana, Javier

Cataldo, Janine K

Catt, Susan

Cavallo, Rossana

Cawthorn, Simon

Ceelen, Wim

Cen, Lin

Ceresoli, Giovanni Luca

Cerutti, Janete

Cervantes, Andres

Cervera, Pascale

Chakraborty, Subhankar

Challacombe, Benjamin J

Chamberlain, Marc

Chan, Andrew T

Chan, Kwok-Wah

Chang, Jeffrey S

Chang, Wen-Wei

Chang-Claude, Jenny

Chanock, Stephen J

Chapman, Judith-Anne

Chatal, Jean-Francois

Chaturvedi, Anil

Chau, Ian

Checkoway, Harvey

Chen, Li Tzong

Cheng, Ann-Lii

Cheng, Iona

Cheng, SK

Cheng, Yee Chung

Cheon, Young Koog

Chia, Kee Seng

Chin, Melvin

Ching, Lai-Ming

Chiniwala, Niyati U

Chintamani, Anupriya

Chlebowski, Rowan T

Chmielecki, Juliann

Choi, Il Ju

Choi, Woonyoung

Chow, Wong-Ho

Christensen, JG

Christophi, Christopher

Chu, Edward

Chua, YJ

Chuang, Shu-Chun

Chung, Y-L

Church, Frank C

Chuu, Chih-Pin

Ciardiello, Fortunato

Cicenas, Jonas

Cioce, Mario

Ciruelos, EM

Clarke, Penny

Clavel, Jacqueline

Clements, Mark

Cnattingius, Sven

Cohen, HT

Cohen, Lorenzo

Colbert, Lisa

Cole, Steve R

Coleman, R

Coley, Helen M

Colleoni, Marco

Collins, E Dale

Collins, Laura

Comstock, Clay ES

Condorelli, Gerolama

Conroy, Shannon M

Cook, Graham P

Cook, Michael B

Cook, Simon J

Cooke, Marcus

Cooksley, Catherine D

Cooper, Colin S

Coppola, Domenico

Coppola, Valeria

Corn, Paul

Corti, Angelo

Cosen-Binker, Laura I

Costagliola, Dominique

Costantini, Elisabetta

Cotte, Eddy

Cottu, Paul H

Coupland, Sarah

Courtice, Midori

Cowling, Victoria H

Cox, Brian

Coyne, James

Cozzi, Renata

Crabb, Simon

Cramp, Fiona

Crawford, SM

Crea, Francesco

Cree, Ian A

Creutzberg, Canien

Crockett, Seth

Crowe, Francesca

Cubie, Heather

Cuezva, Jose M

Cui, Guanglin

Culig, Zoran

Curigliano, Giuseppe

Currow, David C

Cuzick, Jack

Czejka, Martin

Czene, Kamila

Czito, Brian

Dafni, Hagit

D'Agnano, Igea

Dahlin, Anna

Dai, Bingbing

Dal Maso, Luigino

Dalerba, Piero

Dallal, Cher

Dandachi, N

Danesi, Romano

Dang, Chi V

D'Angelica, Michael

Daniel, JM

Darb-Esfahani, Silvia

Darby, Sarah

Darzynkiewicz, Zbigniew

D'Atri, Stefania

Davidson, Susan E

Dawson, Christopher

Day, Matthew

De Azambuja, Evandro

De Bont, Eveline

De Chiara, Loretta

De Gramont, Aimery

De Ioris, Maria Antonietta

De Jong, Daphne

De Jong, Steven

De la Camara, JR

De La Rubia, J

De la Torre, Javier

De Leng, WWJ

De Lima Lopes, Jr, Gilberto

De Maria, R

De Ruysscher, Dirk

De Sanjose, Silvia

De Stefano, Alfonso

De Wever, Olivier

De Wit, Ronald

Dean, Emma J

Dear, Anthony E

Degano, Bruno

DeGraffenried, Linda

Deissler, Helmut

Del Priore, Giuseppe

Delabie, Jan

Delgado, Jorge-Shmuel

Delorme, Stefan

Dempsey, Peter

Demuth, Tim

Deng, Gary

Denham, James W

Denhardt, David T

Denkert, Carsten

Dent, Paul

DeSantis, Evelyn R Hermes

Descourt, Renaud

DeSouza, Leroi

Deutsch, Alexander

Devi, Gayathri R

Dezso, Balazs

Di Fiore, Frederic

Di Lorenzo, Giuseppe

Diamandis, Eleftherios P

Dicato, Mario

Dickman, Paul

Díez Valle, Ricardo

Digiovanna, Michael

Dillner, Joakim

Dillon, Mary

D'Incalci, Maurizio

Ding, Jian

Dingli, David

Dittmer, Dirk P

Dobson, Pauline RM

Dodson, S

Dolcetti, Riccardo

Donato, Rosario

Donington, Jessica

Dontu, G

Dorigo, Oliver

Dou, Q Ping

Douple, EB

Downing, Amy

Downs-Kelly, Erinn

Dowsett, Mitch

Dozin, Beatrice

Dragovich, T

Dragutinovic, Vesna V

Dranitsaris, George

Drapkin, Ronny

Dredge, Keith

Du, Caigan

Du, Xianglin L

Dubeau, Louis

Dübel, Stefan

Dubey, RB

DuBois, Raymond

Dubrova, Yuri

Dubrovska, Anna

Dubsky, Peter

Ducreux, Michel

Duesberg, Peter

Duffau, Hugues

Dumontet, Charles

Dunn, Jeff

Dunn, Sandra

Dusso, Adriana

Dyer, Martin

Dyrskjot, Lars

Eccles, Sue

Eckstein, Niels

Edwards, Claire

Edwards, Dylan R

Eeles, Ros

Eggermont, Alexander

Eilber, FC

Eisenberg, Michael L

Eiser, Christine

Ekbom, Anders

Eklund, Martin

Ekstrom-Smedby, Karin

Elgh, Fredrik

Elias, Anthony

Eliassen, Heather

Elkord, E

Elliot, Sean P

Ellis, Ian

Ellis, Lee M

El-Naggar, Adel

Emmenegger, Urban

Eng, Charis

Enokida, Hideki

Erler, Janine

Ernberg, Ingmar

Erridge, Sara C

Escudier, Bernard

Esteva, Francisco

Esteve, Jacques

Estrela, Jose M

Etzel, Carol

Eva, Lois J

Evans, DGR

Evans, Josie MM

Ewertz, Marianne

Fabbri, Muller

Fabregat, Isabel

Fan, J

Farabegoli, Fulvia

Faratian, Dana

Favoni, Roberto

Fayter, Debra

Fearon, Kenneth CH

Feldman, Darren

Fentiman, Ian

Fernandes, Daniel

Ferreri, Andres

Ferris, Robert L

Field, John K

Figg, William

Figlin, Robert

Filleur, Stephanie

Filmus, Jorge

Fingleton, Barbara

Finke, James H

Fiorica, Francesco

Fischer, Christian

Fitzgerald, David J

Fitzgerald, Rebecca C

Flaherty, Keith

Flaig, Thomas

Flanagan, James

Flowers, LC

Folkerd, Elizabeth

Foran, James

Forbes, Lindsay

Forster, Martin

Fossa, Sophie D

Foster, Claire L

Fotsis, Theodore

Foukakis, Theodoros

Foulkes, William

Fountzilas, George

Fox, Stephen

Franc, Brigitte

Franceschi, Silvia

Francis, David O

Franco, Eduardo

Frank, David

Franklin, Wilbur

Franks, Peter

Frederiksen, Birgitte Lidegaard

Freedman, Neal D

Freeman, Michael

Freifeld, Alison G

Friedenreich, Christine

Fritschi, Lin

Frost, Andra R

Fry, Andrew

Fujita, Ken-ichi

Fujita, Mitsugu

Fukumoto, Manabu

Fulda, Simone

Furuse, Junji

Gabbert, Helmut

Gaffney, D

Galie, Mirco

Galli, Jacopo

Gandhi, Leena

Ganz, Patricia

Garbe, Claus

Garcea, Giuseppe

Garcia del Muro, Xavier

Garcia-Lora, Angel M

Gariboldi, Manuela

Garmyn, Marjan

Garzon, Ramiro

Gasparini, Giampietro

Gatenby, Piers

Gaunt, Piers

Gayther, Simon A

Gebski, VJ

Gelber, Richard

Gemmill, Robert

Gennari, Alessandra

Georgoulias, Vassilis

Gerber, Leah

Gershtein, Elena

Gescher, Andreas J

Ghosh, Paramita

Gianni, Luca

Gietema, JA

Gil Santano, Joan

Giles, Graham

Gill, Sharlene

Gillis, Charles

Gioeli, Daniel

Giorgi Rossi, Paolo

Gires, Olivier

Gluck, Stefan

Gockel, Ines

Goebel, S

Goedert, James

Goel, Ajay

Goetz, Matthew

Goldacre, Michael J

Goldberg, Richard

Goldstein, Mark R

Gomez-Raposo, C

Gondos, Adam

Gong, Liping

Goode, Ellen L

Gopalakrishnan, Vidya

Goppelt-Struebe, M

Gori, Stefania

Gorini, Giuseppe

Goss, K

Goto, Akiteru

Gourley, Charlie

Goy, Andre

Graeler, Marcus

Gramatges, Maria M

Granath, Fredrik

Grant, Steven

Grate, Leslie R

Gray, Peter

Gray, Steven

Grebe, Stefan KG

Green, Jane

Greenhalgh, Joanne

Gregory, Richard I

Grigsby, Perry W

Grimminger, Peter Philipp

Grimson, Peter

Gronberg, Henrik

Grose, Richard

Gross, AL

Gross, Isabelle

Grosso, Anthony

Grote, Karsten

Grothey, Axel

Gruenberger, Thomas

Gu, AD

Guenel, Pascal

Guida, Michele

Guillemette, Chantal

Gunsilius, Eberhard

Guo, Qi

Gurney, James

Gusella, Milena

Gwin, Katja

Gwyther, Steve

Habermann, Thomas M

Hacker, Neville F

Hackshaw, Allan K

Haffty, Bruce

Hagemann, T

Hagen, Anne I

Hague, Angela

Hainsworth, John

Haka, AS

Hakonarson, Hakon

Hakulinen, Timo

Halazun, KJ

Hamada, Jun-Ichi

Hamblin, Michael R

Hamilton, William

Han, Henry

Hancock, Barry W

Hansson, Johan

Hassan, Raffit

Hassan, Y

Hauschild, Axel

Havrilesky, Laura

Hawk, Ernest T

Hayashi, Jun-Ichi

Hayashi, Takuma

Hayes, Andrew

Hayward, Nicholas

He, Jie

He, Yulei

Hedley, David W

Heinzelmann-Schwarz, Viola

Heiss, Markus

Heitz, Florian

Hellman, Asaf

Hemler, ME

Henriksson, Maria

Hensley, Martee

Heracek, Jiri

Heresbach, Denis

Herlyn, Meenard

Hersey, Peter

Hershberger, Patricia E

Hershman, Jerome M

Hertrampf, Katrin

Hess, Georg

Hess, Kenneth R

Hibbitts, S

Hicks, Rodney

Hilakivi-Clarke, Leena

Hildebrand, Janet

Hildebrandt, Gerhard

Hildesheim, Allan

Hillerdal, Gunnar

Hind, Daniel

Hinz, Andreas

Hirano, Takeshi

Hiraoka, Kei

Hochhauser, Daniel

Hoekstra, HJ

Hoffmann, Andreas-Claudius

Hoffmann, Hans

Hohenberger, Peter

Hol, Lieke

Holen, Kyle

Holmberg, Lars

Holmes, Geoffrey K

Holmes, Michelle

Homesley, Howard D

Hood, JL

Hopfner, Michael

Hopp, Nicolás

Horikawa, Izumi

Horst, David

Hoskins, PJ

Houghton, A McGarry

Howard, Louise

Howard, Patricia A

Howe, Louise R

Hsing, Ann W

Hsu, David

Huang, Bin

Huang, Emina H

Huang, Shu-Pin

Huang, Wen-Chin

Huang, Xin

Hubbard, Joleen

Hubbard, Richard B

Huddart, Robert A

Huebschman, Michael

Hugh, Judith

Huguet, Dr Florence

Hummel, Richard

Humphrey, Timothy C

Huntsman, DG

Huq, Fazlul

Hurwitz, Herbert

Husgafvel-Pursiainen, K

Hynes, Nancy

Ibrahim, Ezzeldin

Iggo, Richard

Ignatiadis, Michail

Iinuma, Hisa

Ikeguchi, Masahide

Iljin, Kristiina

Ilson, David

Infante, JR

Ingelsson, Erik

Inoue, Akira

Introna, Martino

Ip, Clement

Islami, Farhad

Isola, Jorma

Itamochi, Hiroaki

Ittmann, Michael

Iversen, PL

Iwazawa, Jin

Izbicki, Jakob R

Jackson, Alan

Jacobs, Bart

Jacot, William

Jagodzinski, PP

Jakowlew, Sonia

Jankowski, Janusz

Janku, Filip

Jansen, Maurice

Janson, Veronica

Janssen-Heijnen, Maryska

Javerzat, Sophie

Jayson, Gordon C

Jazirehi, Ali R

Jefford, Michael

Jenkins, Peter

Jenkins, Valerie

Jensen, Pernille

Jerant, Anthony

Jeronimo, Carmen

Jeys, Lee

Jin, Jianping

Jindal, Rahul M

Jing, Naijie

Joerger, Markus

Johansson, Anna

Jonkers, Jos

Jonsson, Bengt

Jou, TS

Jubb, Adrian

Judson, Ian

Judson, Patricia L

Jukkala, Angela

Jung, Manfred

Kabelitz, D

Kahn, Katherine

Kaijser, Magnus

Kaina, Bernd

Kalaitzakis, EvangelosKalaitzakis

Kalyanaraman, B

Kamangar, Farin

Kanarek, M S

Kanemitsu, Yukihide

Kang, Dongchon

Kang, Yoon-Koo

Kaniwa, Nahoko

Kanopka, Arvydas

Kao, JH

Karapetis, Christos

Karnoub, Antoine

Kashfi, Khosrow

Katalinic, Alexander

Katki, Hormuzd A

Kato, Yukinari

Katoh, Masaru

Kattan, Michael W

Katz, Mira

Keeney, Mike

Keeney, SR

Keep, RF

Keizer, Ron

Kelesidis, T

Kellen, Eliane

Kellokumpu, I

Kelly, Catherine M

Kemeny, Nancy

Kenfield, SA

Kennecke, Hagen

Keshelava, Nino

Kessel, David

Keto, Christopher

Khan, Gulfaraz

Khan, OA

Khan, SA

Khorana, Alok

Kiessling, Fabian

Kikuchi, Shogo

Kim, Clara

Kim, Daniel

Kim, Jae Hoon

Kim, Nayoung

Kim, S

Kim, Sangmi

Kim, Simon

Kim, Sung-O

Kim, Woo Ho

Kinlen, Leo

Kisely, S

Kitajima, Masaki

Kitchener, Henry C

Kivela, Tero

Kleer, C

Klein, Andreas

Klijn, Jan GM

Klymkowsky, MW

Koch, Wayne

Kohno, Kimitoshi

Kohrt, Holbrook E

Kohwi-Shigematsu, Terumi

Komatsu, Yoshito

Koniaris, Leonidas G

Kono, Koji

Konski, Andre

Korfage, Ida

Koroukian, Siran M

Koshiol, Jill

Koskinen, PJ

Koukourakis, Michael

Kourlaba, Georgia

Krajinovic, Maja

Kranc, Kamil

Krikun, Graciela

Krishnamurthy, Savitri

Kristinsson, Sigurdur

Krohn, Kenneth A

Kroll, Mary E

Krouse, Robert S

Krug, Lee

Kruyt, Frank

Krystof, Vladimire

Krzystek-Korpacka, M

Kuhn, DJ

Kuiper, Roland P

Kuipers, Ernst

Kumar, Ashok

Kumar, Rakesh

Kummar, Shivaani

Kunnimalaiyaan, Muthusamy

Kuo, MT

Kurian, Sobha

Kuzmiak, Cherie

Kwan, Marilyn L

Kwon, Janice

La Vecchia, Carlo

La Vine, David BT

Lagergren, Jesper

Lagiou, Pagona

Laiho, Marikki

Lamb, Benjamin

Lan, Michael S

Landberg, Rikard

Landgraf, Ralf

Lane, Rachel

Lange, BJ

Langman, Michael

Larbouret, Christel

Larkin, James

Larsson, Susanna C

Lassen, Ulrik

Law, Malcolm

Lawson, James

Layer, Paul G

Le Tourneau, Christophe

Lebel, S

Ledermann, Jonathan

Lee, AHS

Lee, Hsin-Chen

Lee, MH

Lee, Peter P

Lee, Won Jin

Leggett, Barbara

Leidner, Rom

Leighl, Natasha

Lentzsch, Suzanne

Lesko, Lawrence J

Leslie, NR

Lewis Jnr, JS

Li, Donghui

Li, Jianming

Li, Jingmei

Li, Sonia

Li, Xiao-Nan

Lianidou, ES

Lichtman, Stuart M

Licitra, Lisa

Lièvre, Astrid

Lilja, Hans

Lim, Sung-Chul

Limburg, Paul

Lin, Biaoyang

Lin, Ho

Lin, Ming-Fong

Lin, Shiu-Ru

Lin, Yong

Lindblom, Annika

Lindel, Katja

Linder, Stig

Lindner, Daniel

Lindner, Uri

Lindor, Keith

Lindstrom, Sara

Lipworth, L

Liston, Adrian

Litle, Virginia

Liu, Bolin

Liu, Li

Li-Wan-Po, A

Lo Nigro, Cristiana

Lockwood, WW

Loder, Randall T

Lofgren, Lars

Loi, Sherene

Lonial, S

Lonn, Stefan

Lopes Carvalho, Andre

Lord, Chris

Lord, Sarah

Lordick, Florian

Lorenz, Kai

Loric, Sylvain

Lorincz, Matthew

Lorusso, Vito

Loupakis, Fotios

Low Dog, Tieraona

Lucci, Anthony

Lugli, Alessandro

Lui, W-O

Lukanova, Annekatrin

Lumachi, F

Lund, Bendik

Lund, Leif

Lunt, Sarah Jane

Lutterbach, Bart

Lynge, Elsebeth

Lyratzopoulos, Georgios

Ma, Cynthia X

Mackall, Crystal L

MacKay, Helen

MacLean, Allan B

Macor, P

Maehara, Yoshihiko

Magina, S

Maher, Jane

Majumdar, AP

Mallardo, M

Manfredi, Angelo

Mangili, G

Manjer, Jonas

Mannermaa, Arto

Manning, JT

Mansfield, Aaron S

Mansure, Joao

Mantovani, Alberto

Mantovani, Giovanni

Marais, Richard

Marcos-Gragera, R

Mareel, M

Margulis, Vitaly

Marioni, Gino

Markman, Maurie

Marsit, Carmen J

Martelli, Alberto

Martens, John

Martin, Janet L

Martin, Robert

Martinez-Gonzalez, Jose

Martin-Moreno, JM

Mascitelli, Luca

Mason, Malcolm

Master, Viraj

Masucci, Maria G

Matera, Lina

Matsuo, Keitaro

Matsuo, Koji

Matulonis, Ursula A

Maule, Milena

Mavroudis, D

Maxim, Daniel

Mayer, Arnulf

Mayer, Deborah K

Maynadié, Marc

Mazurak, Vera

Mazzolini, Guillermo

Mazzone, Massimiliano

McAllister, Sean

McAlpine, Jessica N

McArthur, Grant

McCann, Susan E

McConkey, David J

McCubrey, James A

McElroy, Jane A

McEwan, Iain

McGowan, Patricia M

McGurk, Mark

McKay, Siobhan C

McKinnon, Peter J

McMillan, Donald C

McNagny, KM

McNally, Richard

McSheehy, Paul

Medeiros, L Jeffrey

Meijer, Chris JLM

Mele, Donato

Melero, Ignacio

Melichar, Bohuslav

Mell, LK

Menard, Sylvie

Mendonca, Marc S

Menegozzo, Simona

Meng, X

Menigatti, Mirco

Merks, Johannes HM

Mertens, Ann C

Mes-Masson, Anne-Marie

Mezquita, Cristóbal

Michaud, D

Micheau, Olivier

Middleton, Mark R

Midgley, Rachel

Miekisch, Wolfram

Milleron, Bernard

Milne, Christopher

Milne, Roger Laughlin

Minarik, Marek

Ming, Michael

Minna, John

Minton, Ollie

Mir, Olivier

Mishra, Rajakishore

Mittendorf, Elizabeth A

Miyazaki, Kohji

Modlin, Irvin M

Moebus, Volker

Mogensen, Mette M

Mok, Samuel C

Mok, Tony

Molinari, Michele

Moller, Henrik

Molleví, DG

Mols, Floortje

Momparler, Richard L

Monk, Bradley

Monsees, Genevieve

Montano, Monica M

Montazeri, Ali

Montgomery, David Andrew

Moore, Richard G

Morabito, Fortunato

Moradi, Tahere

Morandi, Veronica

Moreau, Philippe

Moreno, Lucas

Morgan Jr, Robert J

Morgan, Bruno

Morgan, Todd

Morice, Philippe

Morii, Eiichi

Morita, Masaru

Morris, LG

Morris, PG

Morrison, David

Morton, Lindsay

Mosser, Jean

Moulder, Stacy

Mowla, SJ

Mross, Klaus

Mueller-Klieser, Wolfgang

Muggia, Franco

Muir, Kenneth

Mukherjee, Somnath

Mulatero, Clive

Mulder, Karen

Mulder, Nanno H

Muller, David

Mulrooney, Daniel A

Murphy, Gillian

Nagel, Gabriele

Nagourney, Robert A

Nahta, Rita

Nakabeppu, Y

Nakajima, Atsushi

Nakashima, Jun

Nam, Joo-Hyun

Narod, Steven A

Natsume, Atsushi

Nawrocki, Steffan

Neal, Joel W

Negri, Eva

Neklason, Deborah

Nelson, Gill

Neoptolemos, John

Neri, Dario

Neuwelt, Edward A

Newton, H

Newton, Robert

Neyroz, Paolo

Nguyen, David

Niclou, Simone P

Nicolini, Andrea

Nie, Daotai

Nielsen, Hans Jorgen

Nielsen, Torsten O

Nilsonne, Gustav

Nislow, Corey

Nitti, Donato

Niu, Xiaoling

Niwinska, Anna

Noble, Dr Simon

Nohe, Anja

Nordlinger, Bernard

Norman, Paul

Normanno, Nicola

Nothlings, Ute

Nur, Ula

Nurmi, Tarja

Nyren, Olof

Nystrom, Lennarth

Obermajer, Natasa

O'Byrne, Kenneth J

Ochiya, Takahiro

O’Donnell, Peter H

O’Donovan, Norma

O’Dwyer, Peter J

O’Flaherty, Joseph

Ofner, Clyde

Ogawa, Kazuhiko

Ogino, Shuji

Ognjanovic, Simona

Ohnishi, Hirohide

Okabe-Kado, Junko

Okines, Alicia

Olmos, David

Olson, Janet E

Omuro, Antonio M

Ondrusova, Martina

O'Reilly, Eileen

Orntoft, Torben F

O'Rorke, Michael

Ortega-Deballon, Pablo

Ortmann, Olaf

Osborne, Richard

Oscarsson, Marie

Ott, Patrick

Ouadid-Ahidouch, Halima

Owen, David W

Oza, Amit

Ozmen, Vahit

Padhani, Anwar

Paesmans, Marianne

Pajonk, Frank

Palacios, Jose

Palmer, Daniel H

Palmieri, Giuseppe

Palombo, Fabio

Pandita, Tej

Pantel, Klaus

Paoletti, Xavier

Paraskeva, Chris

Pardo, Luis

Parente, F

Park, In Hae

Park, Jong Y

Park, Sook Ryun

Park, Sue K

Parkin, Donald Max

Parkinson, Eric Kenneth

Parola, Maurizio

Parrella, Paola

Parulekar, Wendy

Pasche, Boris

Pascoe, Jennifer

Pasheva, Evdokia

Patil, Sujata

Paulson, James C

Paulus, S

Peake, Mick

Pedley, R Barbara

Peggs, Karl

Peidis, P

Pejovic, Tanja

Pelicci, Giuliana

Pencavel, Tim D

Penney, Kathryn L

Perez-Soler, Roman

Perez-Stable, Carlos

Perrone, Francesco

Perrotti, Danilo

Pestalozzi, Bernhard

Peters, Jeffrey M

Peters, Ulrike

Petersen, LJ

Petignat, Patrick

Petridou, Eleni

Petrik, Jim

Petru, Edgar

Phang, James M

Pharoah, Paul DP

Phelan, CM

Philip, Philip

Phillips, Wayne

Phipps, Al

Phipps, Etienne

Piché, Alain

Pickard, A Simon

Pierga, Jean-Yves

Pili, Roberto

Pinto, Carmine

Pinto, Ligia A

Pisani, Paola

Pischon, Tobias

Pishvaian, Michael J

Plöckinger, Ursula

Plukker, John ThM

Poch-Broto, Joaquín

Poirier, Guy G

Pole, Jason

Pollan, M

Pompella, A

Pope, WB

Porfiri, Emilio

Powell, Matthew A

Pozzi, Ambra

Prabhala, Rao

Prasad-Hayes, Monica

Price, Timothy

Prince, ME

Pringle, J Howard

Pritchard, Kathleen I

Procopio, Antonio

Proctor, Michael

Protti, Maria Pia

Prudkin, Ludmila

Puccetti, Paolo

Puente, Xose S

Pukkala, Eero

Purrello, Michele

Quaglia, Alberto

Quinn, Gwen

Quoix, Elisabeth

Rabkin, Charles S

Rahman, Nazneen

Rajasekaran, AK

Rampling, Roy

Ramroth, Heribert

Rao, Sheela

Rapley, Elizabeth A

Rathmell, W Kimryn

Ratnam, Manohar

Ratner, Desiree

Ravaud, Alain

Ray, Ramesh M

Raynaud, Florence

Rayson, Daniel

Rédini, Françoise

Redman, Mary

Reed, Gregory A

Reedijk, Michael

Rees, Myrddin

Reeves, Gillian K

Reichardt, Peter

Reis, Flávio

Reis-Filho, Jorge

Renehan, Andrew G

Renwick, William

Reulen, Raoul C

Reynolds, John V

Ricciardiello, Luigi

Richardson, Des R

Rickles, Frederick R

Riganti, Chiara

Rigas, Basil

Rimassa, Lorenza

Ring, Alistair E

Rini, Brian

Roach, Mack

Robb, Kathryn

Robert, Jacques

Robertson, Erle S

Robertson, John M

Robinson, Dominic J

Robson, Mark E

Rocca, Walter

Roddam, Andrew W

Rodenhuis, Sjoerd

Rodríguez-Berriguete, Gonzalo

Rodríguez-López, José Neptuno

Roger, Sébastien

Romanska, Hanna

Roperto, S

Rosati, Emanuela

Rosch, Paul

Rosen, Alexandre

Rosenthal, Adam

Rosenthal, T

Ross, Paul J

Ross, Richard

Rossig, Claudia

Rossmeisl, JH

Roupret, Morgan

Rouzier, Roman

Rubinstein, Larry

Ruder, Liz

Rudlowski, Christian

Ruol, Alberto

Rustin, Gordon JS

Ryan, Paula

Ryden, Lisa

Saad, Fred

Sadikovic, Bekim

Safe, Stephen

Safran, Howard

Saha, Tapas

Sainsbury, Richard

Saito, Kazutaka

Sala, Arturo

Saldhana, Gerald

Salto-Tellez, Manuel

Salz, Talya

Sanchez-Gomez, Myriam

Sandblom, Gabriel

Sandin, Sven

Sandri, Maria Teresa

Sankaranarayanan, R

Sanson, Marc

Sant, Milena

Sapino, Anna

Sardi, Iacopo

Sarkadi, Balazs

Sautès-Fridman, Catherine

Sawada, Tokihiko

Scarpa, Aldo

Scartozzi, Mario

Schafer, Beat

Scheller, Jürgen

Schelman, WR

Schemshedini, L

Scheper, Rik J

Schiemann, WP

Schiffman, Mark

Schlaepfer, DD

Schleiermacher, Gudrun

Schlemmer, Marcus

Schlumberger, Martin

Schmeler, Kathleen M

Schmidt, Marjanka K

Schmoll, Hans-Joachim

Schmutzler, Rita K

Schoennemann, KR

Schonfeld, Sara

Schootman, Mario

Schouten, Leo J

Schuz, Joachim

Schwartz, David L

Schwartz, EL

Schwartz, Gordon

Schwartz, Stanley A

Scorilas, Andreas

Scotet, Emmanuel

Sekido, Yoshitaka

Sekine, Ikuo

Seliger, Barbara

Semiglazov, Vladimir

Senner, Volker

Seno, Masaharu

Senore, Carlo

Seshadri, Mukund

Sessa, Cristiana

Setaluri, Vijayasaradhi

Sevdalis, Nick

Shah, Keerti

Shammas, Masood

Shamseddine, Ali

Sharabi, Amir

Sharma, RA

Sharma, Sangita

Sharpley, CF

Shaw, Jacqueline A

Sheahan, Kieran

Shelley, Mike

Shenfine, Jon

Shepherd, John A

Shibata, Tatsuhiro

Shiels, MS

Shigehara, Kazuyoshi

Shimoyama, Shouji

Shiozawa, Manabu

Shipley, Janet

Shukla, Y

Siassi, Michael

Sicklick, Jason

Sidhu, Stan

Siefker-Radtke, Arlene

Siena, Salvatore

Sieuwerts, A

Silliman, Rebecca

Silver, Andrew

Silverberg, Michael J

Silverman, Barbara

Simard, Julia

Simon, Alice

Simpkins, Henry

Simpson, Evan

Simpson, Peter T

Singer, Christian

Singer, Susanne

Singh, Gurmit

Singh, H

Singh, Piksi

Singh, Rajan

Singhal, Sharad

Sinicrope, Frank

Sipahi, Ilke

Skipworth, Richard JE

Slaby, O

Slavutsky, Irma

Sleijfer, Stefan

Small, William

Smalley, Keiran SM

Smith, Paul J

Smith-McCune, K

Smyth, Mark

Soini, Ylermi

Soliman, Hatem

Somji, Seema

Sommers, Benjamin

Sonnenberg, A

Sonpavde, Guru

Sorà, Federica

Soria, Jean-Charles

Sousa, Hugo

Spandidos, Demetrios A

Sparen, Par

Spring, Kevin J

Stacchiotti, Silvia

Stal, Olle

Stallings, Raymond

Stamp, Gordon

Stanbridge, Eric

Stanford, Janet L

Stang, Andreas

Stankov, Karmen

Stead, Lucy

Stebbing, Justin

Steel, C Michael

Steele, Nicola

Steele, Robert

Stegh, Alexander H

Steliarova-Foucher, E

Stemmler, Joachim

Stephens, Richard

Stern, Marc-Henri

Stern, Mariana

Stern, Peter

Steward, William P

Stewart, Colin J

Stewart, Fiona A

Stokoe, David

Stolzenberg-Solomon, Rachael

Stone, Nicholas

Stoppa-Lyonnet, Dominique

Storey, Dawn

Stournaras, Christos

Stratakis, Constatine A

Streubel, Berthold

Strickler, Howard

Ströhlein, Michael A

Sturge, Justin

Suda, Toshio

Sukumvanich, Paniti

Sun, Minghao

Sundar, Sudha

Sundberg, Carl Johan

Supriyadi, E

Sutherland, Robert L

Sutton, Gregory

Suva, L

Svane, Inge Marie

Swanton, Charles

Sweetenham, John

Szallasi, Zoltan

Szlosarek, Peter

Szoke, Tamas

Szpechcinski, Adam

Tagliabue, Elda

Taioli, Emanuela

Takahashi, I

Takeuchi, Hiroya

Takigawa, Nagio

Talbott, Evelyn O

Tamimi, Rulla M

Tamura, Tomohide

Tan, Winston

Tanaka, Fumiaki

Tanaka, Kazuhiro

Tanaka, Kimio

Tanaka, Shinji

Tanji, Nozomu

Tattersall, Martin HN

Tavani, Alessandra

Taylor, Carolyn

Taylor, Kathryn

Taylor, Sally

Tebbutt, Niall Christopher

Teglund, Stephan

Teh, Bin

Tentes, Ioannis

Tewari, M

Thirlwell, Chrissie

Thomas, Anne

Thomas, Gareth J

Thompson, John A

Thorat, Mangesh

Thorne, Heather

Thorne, Steve

Thuss-Patience, Peter

Tinker, Anna

Tlsty, Thea D

Todo, Yukiharu

Toffoli, Giuseppe

Tol, J

Tolaney, Sara

Tolcher, Anthony W

Tombal, Bertrand

Tonini, Giuseppe

Torp, Steffen

Torres-Mejía, Gabriela

Torres-Roca, Javier

Torta, Riccardo

Touze, Antoine

Townsend, Paul A

Treasure, Tom

Trpkov, Kiril

Trufero, Javier

Tryggvadottir, Laufey

Tseng, Ling-Ming

Tsugane, Shoichiro

Tsushima, Yoshito

Tulchinsky, E

Tung, Nadine

Turner, Robin

Tworoger, Shelley

Tynninen, Olli

Tzanakakis, George

Uchiumi, Takeshi

Udono, H

Uhr, Jonathan

Uramoto, Hidetaka

Urayama, Kevin Y

Ushijima, Kimio

Usuda, Jitsuo

Uzawa, Katsuhiro

Vacek, Pamela

Vachon, Celine

Valdimarsdottir, Unnur

Vallance, Patrick

Valle, Juan W

Van der Speeten, Kurt

Van der Veldt, Astrid

Van Hemelrijck, Mieke

Van Laar, M

Van Leerdam, Monique

Van Tellingen, Olaf

Van Waarde, Aren

Van Weert, Julia CM

Vasiliou, Vasilis

Vasiljeva, Olga

Vassella, Erik

Vauthey, Jean-Nicolas

Velikova, Galina

Verbeek, JHAM

Vereb, György

Vergote, Ignace

Verheij, Marcel

Verschraegen, Claire

Verweij, Jaap

Vincent, Mark

Vincenzi, Bruno

Vinogradova, Yana

Vivanco, Maria

Voduc, K David

Vokes, Everett

Von Minckwitz, Gunter

Von Wagner, Christian

Vousden, Karen

Wabinga, Henry

Wacholder, Sholom

Wagner, Oswald

Wainberg, Zev

Wakeford, Richard

Waller, Jo

Walsh, Naomi

Walsh, Paul M

Wang, Enhua

Wang, Hsei-Wei

Wang, Jin

Wang, Kai

Wang, Li

Wang, Ming-Rong

Wang, Qianben

Wang, Tian-Li

Warburton, D

Warde, Padraig

Warmuth, Marisa

Wasserfallen, Jean-Blaise

Watanabe, Y

Watkins, DK

Weigelt, Britta

Weinberg, Armin

Weis, Joachim

Weisberg, Ellen

Weissfeld, Joel

Welch, Stephen

Welinder, Charlotte

Weller, Michael

Welsh, Chris

Weng, Ching-Feng

Wesa, Kathleen

Wesseling, J

West, Catharine ML

Westbrook, Thomas F

Westermarck, J

Westin, Shannon

Wheatley, Keith

White, Emily

White, Jeffrey D

White, Rebekah R

Whitman, Gary

Widmer, Nicolas

Willett, Christopher

Williams, Elizabeth

Williams, Gareth Haydn

Williams, Graeme

Williams, Scott G

Wilop, Stefan

Wilschut, Janneke

Wilson, Shandra

Winer, Rachel

Winquist, Eric

Woldemichael, GM

Wolpin, Brian M

Wong, Germaine

Wong, Kwong-Kwok

Woolgar, J

Wouters, An

Wright, Jason D

Wright, Jonathan L

Wright, Margaret E

Wroblewski, Lydia

Wu, Lijun

Wyld, Lynda

Wyllie, Andrew

Xenocostas, Anargyros

Xie, Y

Xu, Guowang

Xu, Liang

Xu, XX

Xue, Chengyuan

Xue, Lexun

Yamamoto, Hidetaka

Yamashita, Hiroharu

Yang, Chih-Hsin

Yang, Wei

Yang, Wen Lang

Yang-Yen, Hsin-Fang

Yankeelov, Thomas

Yau, Thomas

Ye, Weimin

Yeh, Kun-Yun

Yen, Yun

Yilmaz, Yusuf

Ylikorkala, Olavi

Yoshioka, Takashi

Young, Scott D

Yu, Herbert

Yu, J

Yuspa, Stuart H

Zafar, S Yousuf

Zaffaroni, Nadia

Zafrakas, M

Zain, Jasmine

Zaino, Richard

Zajac, Ian T

Zalaudek, Iris

Zhang, Bin

Zhang, Fang Fang

Zhang, Wei

Zhang, Xiaohui

Zhang, Xuehong

Zhao, Hong

Zheng, Qi-Huang

Zhou, Jinyuan

Zhu, Yong

Ziogas, Dimosthenis

Zur Hausen, Axel

